# Relationship between seasonal cold acclimatization and mtDNA haplogroup in Japanese

**DOI:** 10.1186/1880-6805-31-22

**Published:** 2012-08-28

**Authors:** Takayuki Nishimura, Midori Motoi, Yousuke Niri, Yoshikazu Hoshi, Ryuichiro Kondo, Shigeki Watanuki

**Affiliations:** 1 Graduate School of Design, JSPS Research Fellow DC, Kyushu University, Fukuoka, 815-8540, Japan; 2 Graduate School of Integrated Frontier Sciences, Kyushu University, Fukuoka, 812-8581, Japan; 3 School of Agriculture, Tokai University, Kumamoto, 869-1404, Japan; 4 Department of Forest and Forest Products Sciences, Faculty of Agriculture, Kyushu University, Fukuoka, 812-8581, Japan; 5 Department of Human Science, Faculty of Design, Kyushu University, Fukuoka, 815-8540, Japan

**Keywords:** Cold adaptation, Seasonal cold acclimatization, mtDNA haplogroup, Cold exposure, Oxygen consumption

## Abstract

**Background:**

The purpose of this study was to elucidate the interaction between mtDNA haplogroup and seasonal variation that contributes to cold adaptation.

**Methods:**

There were 15 subjects (seven haplotype D subjects and eight haplotype non-D subjects). In summer and winter, the subjects were placed in an environment where the ambient temperature dropped from 27 °C to 10 °C in 30 minutes. After that, they were exposed to cold for 60 minutes.

**Results:**

In summer, the decrease in rectal temperature and increase in oxygen consumption was smaller and cold tolerance was higher in the haplotype non-D group than in the haplotype D group. In winter, no significant differences were seen in rectal temperature or oxygen consumption, but the respiratory exchange ratio decreased in the haplotype D group.

**Conclusions:**

The results of the present study suggest that haplogroup D subjects are a group that changes energy metabolism more, and there appears to be a relationship between differences in cold adaptability and mtDNA polymorphism within the population. Moreover, group differences in cold adaptability seen in summer may decrease in winter due to supplementation by seasonal cold acclimatization.

## Background

Cold adaptation in humans has long been debated. Modern humans (*Homo sapiens*) spread from Africa to all parts of the world, and it is thought that, in the process, they adapted to various environments, particularly to cold climates, using various strategies. Among these strategies there are different types of cold adaptation that vary with factors such as strength of cold stimulation, food situation, and cultural background. These types of adaptation include: isolative adaptation, in which a person exposed to a constant level of cold exhibits a decrease in skin temperature without a change in core body temperature [1-3]; hypothermic adaptation, in which a person exhibits lower core body temperature [4,5]; isolative hypothermic adaptation, in which a person exhibits both a decrease in skin temperature and lower core body temperature [6,7]; and metabolic adaptation, in which thermogenesis is strengthened [8,9]. These adaptation types are often interpreted as regional characteristics of a population that has been in a certain environment for a long time and may also include genetic adaptations.

When humans are exposed to cold, they exhibit physiological responses to maintain their body temperature. The first response is for blood vessels to constrict and suppress heat loss from the skin surface. In thermoregulation by vasoconstriction, the range of controllable temperature (thermoneutral zone) is small. The response to even colder stimulation is energy metabolism, including shivering thermogenesis (ST) and nonshivering thermogenesis (NST). Differences in physiological responses arise depending on differences in the type of adaptation. Examples of this difference are individuals with strong heat insulation ability due to vasoconstriction and individuals who exhibit an early response via metabolism. These variations are not considered statistical errors, but rather physiological differences or physiological polytypisms, resulting from individual differences in adaptation strategy. In addition to sex and age, these differences are also influenced by environmental factors associated with aspects such as season, lifestyle habits, genotype, and other genetic factors that act as the base, while these factors are also associated with morphological characteristics [10-12]. The type of physiological response to cold adaptation is thought to be largely built on the interaction between environmental and genetic factors. However, few studies have focused on the genetic factors.

In short-term cold acclimatization studies during which subjects were exposed to cold stimulation in an artificial climate chamber with a room temperature of 5 °C or were immersed in cold water, the heat insulation capacity was reportedly enhanced by a reduction in blood flow following acclimatization associated with a decrease in thermogenesis, a delay in shivering, and a decrease in distal skin temperature [13,14]. In contrast, no changes were seen in core body temperature and mean skin temperature from cold acclimation after cold exposure in a 5 °C room for 2 hours per day for 11 days [15]. According to different studies on relatively long seasonal acclimatization, after acclimatization in winter the shivering and thermogenesis were reported to decrease [2], show no change [16-18], or increase [19-22]. Skin temperature has also been reported to decrease [15], show no change [20], or increase [21,22]. Cold acclimatization in humans is thus reported to have a seasonal component, but there is currently no consensus on the nature of this component. In addition to individual differences, this component may vary with the level of cold stimulation depending on factors such as exposure conditions and amount of clothing. Cold water immersion and other types of strong cold stimulation are often reported to result in a decrease in skin temperature and a decrease in thermogenesis [13,14], suggesting that the strength of the temperature environment may also affect the population in studies on seasonality because the strength of cold stimulation depends on their area of habitation.

Few studies have explained genetic factors related to the cold tolerance response, adaptation, and acclimatization. Generally speaking, morphological differences in mammals among different populations of the same species are based on genetic factors, as specified by Allen’s rule and Bergmann’s rule. From a physiological perspective, metabolic adaptations that rely on a higher basal metabolic rate or thermogenesis, such as those seen in Inuit people, are thought to be genetic adaptations [23], but the underlying mechanism is not yet clear. According to recent reports, mitochondria and their genomes that may influence genetic factors for cold tolerance may partially elucidate this mechanism [24-26]. Mitochondria are the basis for energy metabolism, which serves an important role in the cold tolerance response. The thermogenic response to cold stimulation includes ST by skeletal muscles and NST by internal organs and brown adipose cells, and thermogenesis is performed in these tissues and cells through mitochondria. In many previous studies, changes in the amount of thermogenesis (metabolic rate, oxygen consumption) have been argued to depend on the presence or absence of shivering [2,13,14]. However, recent studies have reported an increase in thermogenesis even in conditions when shivering does not occur [21,22], and they have suggested that NST is involved in cold tolerance [27-29]. The latest studies have shown that brown adipose cells also become activated by cold stimulation in adults, and they may play a part in thermoregulation [30]. According to another report, brown adipose cells are highly active in winter and their activity declines as humans age [31]. Uncoupling protein is also present in mitochondria in skeletal muscles, suggesting that NST takes place there [32,33]. Thoughts on ST and NST have thus changed, and variation within single individuals has been suggested.

As mitochondria have their own genome, this genome may influence functional differences in mitochondria. Because of their evolutionary neutrality, the mitochondrial genome is an important means of understanding human migrations with accompanying age estimates [34]. In recent reports, other researchers have claimed that adaptations to climate have been made by mtDNA regulating the balance of ATP generation and thermogenesis in oxidative phosphorylation of mitochondria [24-26]. Thermogenesis here is not that produced by cold stimulation, but rather by cellular-level thermogenesis released during ATP generation. The principle is similar to that of an engine, in which the process of generating energy from raw materials is never 100% efficient, with some heat always escaping. While the efficiency of ATP generation varies depending on the conditions [25], it has been suggested that mitochondria of populations that have adapted to cold have a basal metabolism that generates heat more easily, so the influence of thermogenesis is greater, even with the same amount of oxygen consumption. This means that a large volume of heat may be obtained in states such as ST and NST and basal metabolism where oxygen is consumed. In relation to this hypothesis, it has often been reported that mtDNA polymorphism influences physiology in modern humans, mostly via energy metabolism systems. For example, maximum oxygen consumption is small in haplogroup J people [35], there are differences between haplotypes in the risk of developing diabetes or other lifestyle-related diseases that are closely related to energy metabolism [36], there is an association between basal metabolism and mtDNA polymorphism [37], and there is an association between acute altitude sickness and mtDNA polymorphism [38], In cold adaptation studies carried out by our research group in summer [39], people with haplogroup D – the most common haplotype in Japanese people – showed the same amount of oxygen consumption during cold exposure but a smaller decrease in rectal temperature compared with haplogroup non-D people. The results of this study suggested that mtDNA polymorphism is one factor that causes variation in cold tolerance.

While the above findings suggest that genetic factors have some sort of influence on seasonal acclimatization and acclimatization to repetitive exposure, this has yet to be argued. In particular, there is a need to investigate how genetic factors affect differences in physiological responses between summer, during which there is no cold acclimatization, and winter, during which there is cold acclimatization. There are different arguments about how to define cold tolerance. In the present study, high cold tolerance was defined as a small decrease in core body temperature in response to an increase in energy metabolism. This definition was used because, during cold stimulation that exceeds the zone for insulation thermoregulation by skin vasoconstriction, an increase in energy metabolism is the only means for thermoregulation, and the relationship between energy metabolism and core body temperature becomes very important. Theoretically, a cold tolerance response that relies on inherent genetics is predicted to play a large role in maintaining body temperature in summer and a smaller role in winter due to cold acclimatization. The reason for this difference may be adaptive seasonal variation from summer to winter that has been reported in many previous studies. More specifically, if mtDNA polymorphism is involved in energy metabolism, it may play some part in determining whether metabolic adaptation is exhibited or isolative adaptation is exhibited in response to an equal amount of stimulation. The present study thus focused on mtDNA polymorphism and aimed to elucidate how changes in cold tolerance and season occur with the interaction between the seasonal cold acclimatization and haplogroup. While the sample size may be too small to determine genetic effects, the same methods were used in a study conducted in the summer, and the same subjects were examined to control for body mass index and body surface area as much as possible.

## Methods

### DNA analysis

Total DNA was extracted from hair shafts by digestion in extraction buffer using ISOHAIR (Code No. 319–03401; Nippon Gene, Tokyo, Japan.

The mtDNA gene spacer D-loop was amplified by PCR using primers M13RV-L15996 and M13(−21)-H408. The analyzed sequences of the D-loop primers were as follows: mtDNA L15996, 5′-CTCCACCATTAGCACCCAAAGC-3′; and mtDNA H408, 5′-CTGTTAAAAGTGCATACCGCCA-3′.

The thermocycling profile consisted of an initial denaturation step at 94 °C for 1 minute, followed by 32 cycles for 30 seconds at 94 °C, 30 seconds at 56 °C, and 75 seconds at 72 °C. Purified DNA was sequenced in both directions using the ABI PRISM 310 Genetic Analyzer (Applied Biosystems, Foster City, CA, USA) with a BigDye Terminator v3.1 Cycle Sequencing Kit (Applied Biosystems).

### Participants

To find genetic effects, variations in morphological characteristics within subjects were minimized, since cold adaptability depends on morphological characteristics. Participants were divided into haplogroup D (D4) and haplogroup non-D (not common in the northern zone) because the number of participants was limited in this experiment. A total of 15 subjects who participated in the cold exposure experiment, including seven haplotype D (D4) students and eight students of haplotype non-D, were selected so that there were no significant differences in morphological characteristics (height, weight, body mass index, body surface area, body fat), as shown in Table 1. The haplogroups of non-D subjects were M7a (four subjects), M7c (one subject), F2a (one subject), and B4 (two subjects). Body surface area was calculated by Kurazumi’s formula [40], and body fat was calculated by Brozek’s formula [41]. The subjects were born in Fukuoka Prefecture or neighboring prefectures, and they did not include any individuals who participated regularly in vigorous sports. The mtDNA analysis was performed with approval from the Ethics Committee for Genome-gene Analysis of the Graduate School of Medicine, Kyushu University. In addition, mtDNA information obtained in our study was anonymously treated and managed by the Gene Therapeutic Information Center of the university.

**Table 1:** Subjects’ morphological characteristics

**Season**	**Haplotype**	**Height (cm)**	**Weight (kg)**	**Body mass index**	**Body surface area (m**^**2**^**)**	**Body fat (%)**
Summer	D (*n* = 7)	172.7 ± 5.8	62.4 ± 5.6	20.8 ± 1.8	1.73 ± 0.09	13.6 ± 2.2
	Non-D (*n = 8*)	171.1 ± 4.1	59.1 ± 4.0	20.2 ± 1.1	1.69 ± 0.06	14.1 ± 2.0
Winter	D (*n* = 7)	173.1 ± 5.6	61.2 ± 5.7	20.4 ± 1.8	1.72 ± 0.08	13.3 ± 2.4
	Non-D (*n* = 8)	171.8 ± 3.9	59.3 ± 4.6	20.1 ± 1.1	1.70 ± 0.07	14.4 ± 2.3

### Measurements

The experiments were conducted in Fukuoka during summer (August to September) and winter (February to March), and the mean temperature in summer was 29.0 °C and in winter was 8.5 °C. Measurement sensors were attached to subjects in an environment with a temperature of 27 °C in preparation for the experiment. The subjects rested quietly for 15 minutes in an artificial climate chamber, and then the cold exposure commenced. The artificial climate chamber was programmed so that the ambient temperature dropped to 10 °C in approximately 30 minutes, after which there was exposure to cold (10 °C) for 60 minutes.

The parameters recorded were rectal temperature, skin temperature (seven places), oxygen consumption, blood pressure, electrocardiogram, and a subjective evaluation. The rectal temperature probe was inserted to a depth of 13 cm beyond the anal sphincter. The skin temperature sensors were attached with surgical tape to measurement sites on the forehead, shoulder, chest, forearm, back of the hand, thigh, and dorsal side of the foot. Measurements were made continuously at intervals of 2 seconds using a data logger (LT-8A; Gram Corporation, Saitama, Japan). Mean skin temperature was calculated from the seven-point method of Hardy–DuBois[42]. Distal skin temperature was derived using the following equation involving arm, hand, feet and leg temperatures :

(1) $$\text{Distal skin temperature}=(0.14\times T_{\text{arm}}+0.05\times T_{\text{hand}}+0.07\times T_{\text{feet}}+0.13\times T_{\text{leg}})/0.39$$

The weighting factor for each body segment was based on Hardy and Du Bois, 0.39 being the relative surface area of the distal and proximal regions, respectively. Oxygen consumption was measured with a respiratory gas analyzer (AE-300S; Minato Medical Science, Osaka, Japan) through a breathing tube using a mask to measure expired gas (Rudolph mask; Nihon Kohden, Tokyo, Japan).

### Statistical analysis

Morphological data were compared by unpaired *t* test. Physiological data were compared using three-way (haplogroup and season and time) analysis of variance. All data are expressed as the mean ± standard error, and *P <* 0.05 was considered significant.

## Results

### Rectal temperature

**Figure 1:**
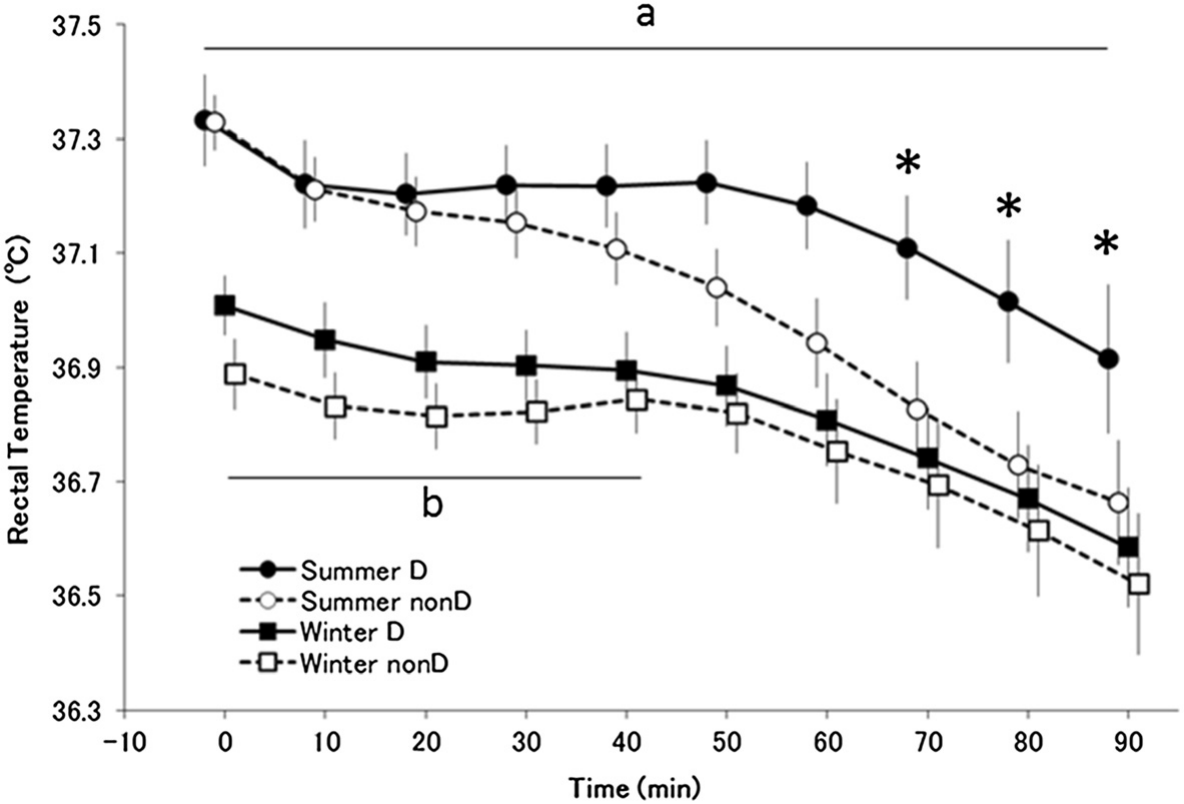
**Rectal temperature.** Changes in rectal temperature (means ± standard error) in summer D (●; *n* = 7), summer non-D (○; *n* = 8), winter D (■; *n* = 7), and winter non-D (□; *n* = 8) groups. * *P <* 0.05 compared with summer D (●) and summer non-D (○), ^a^*P <* 0.05 compared with summer D (●) and winter D (■), and ^b^*P <* 0.05 compared with summer non-D (○) and winter non-D (□). In a *post hoc* test, rectal temperatures at rest (0 min) were lower in winter than in summer for both groups. Rectal temperatures of haplogroup D subjects when exposed to cold during winter were significantly lower at all time points than exposure during summer ( *P <* 0.05). In contrast, non-D subjects had significantly lower rectal temperatures 0 to 40 minutes after the start of exposure compared with summer ( *P <* 0.05), but no seasonal difference was seen after 50 minutes. In summer, haplogroup D subjects had higher rectal temperatures than haplogroup non-D subjects 70, 80, and 90 minutes after the start of cold exposure ( *P <* 0.05)

Analysis of variance revealed significant differences in the main effect of season (*F*_(1,13)_ = 14.50, *P <* 0.005) and the main effect of time ( *F*_(9,117)_ = 36.63, *P <* 0.001) for rectal temperature (Figure 1). The interaction of season and time was significant (*F*_(9,117)_ = 2.383, *P <* 0.05). The interaction of season and haplogroup and time was also significant ( *F*_(9,117)_ = 5.168, *P <* 0.001).

In a *post hoc* test, rectal temperatures at rest (0 minutes) in a 27 °C room were lower in winter than in summer for both groups. Rectal temperatures of haplogroup D subjects when exposed to cold during winter were significantly lower at all time points than when exposed during summer ( *P <* 0.05). In contrast, non-D subjects had significantly lower rectal temperatures 0 to 40 minutes after the start of exposure compared with summer ( *P <* 0.05), but no significant seasonal difference was seen after 50 minutes. In summer, haplogroup D subjects had higher rectal temperatures than non-D subjects 70, 80, and 90 minutes after the start of cold exposure ( *P <* 0.05).

### Changes in rectal temperature

**Figure 2:**
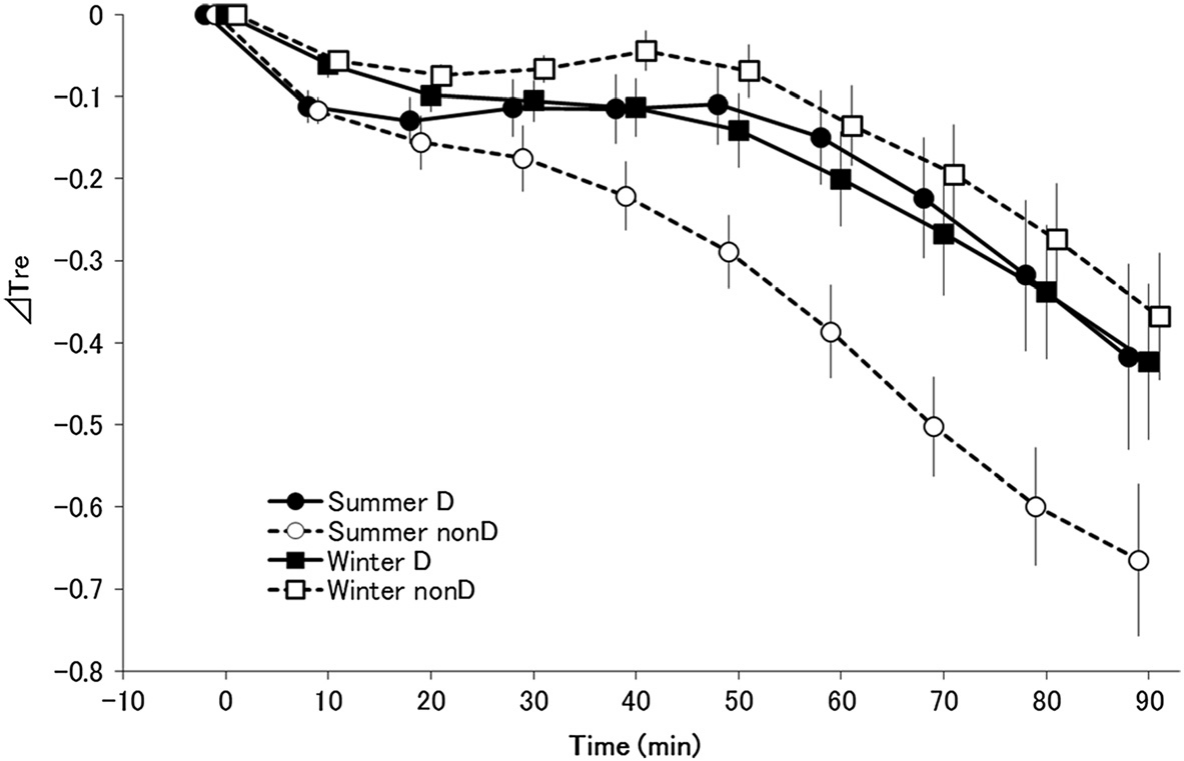
**Change in rectal temperature.** Changes in rectal temperature (ΔT_re_; mean ± standard error) in summer D (●; *n* = 7), summer non-D (○; *n* = 8), winter D (■; *n* = 7), and winter non-D (□; *n* = 8) groups. In a *post hoc* test, rectal temperatures were significantly lower from 40 minutes after the start of cold exposure only in the summer non-D group ( *P <* 0.05)

The main effects of season (*F*_(1,13)_ = 6.236, *P <* 0.05) and time ( *F*_(9,117)_ = 36.609, *P <* 0.001) were significant for changes in rectal temperature (Figure 2). There was a significant interaction between group and season (*F*_(1,13)_ = 7.106, *P <* 0.005) and between season and time ( *F*_(9,117)_ = 2.376, *P <* 0.05). The interaction among group, season, and time was also significant ( *F*_(9,117)_ = 5.170, *P <* 0.001).

In a *post hoc* test, rectal temperatures were significantly lower from 40 minutes after the start of cold exposure only in the summer non-D subjects ( *P <* 0.05).

### Oxygen consumption

**Figure 3:**
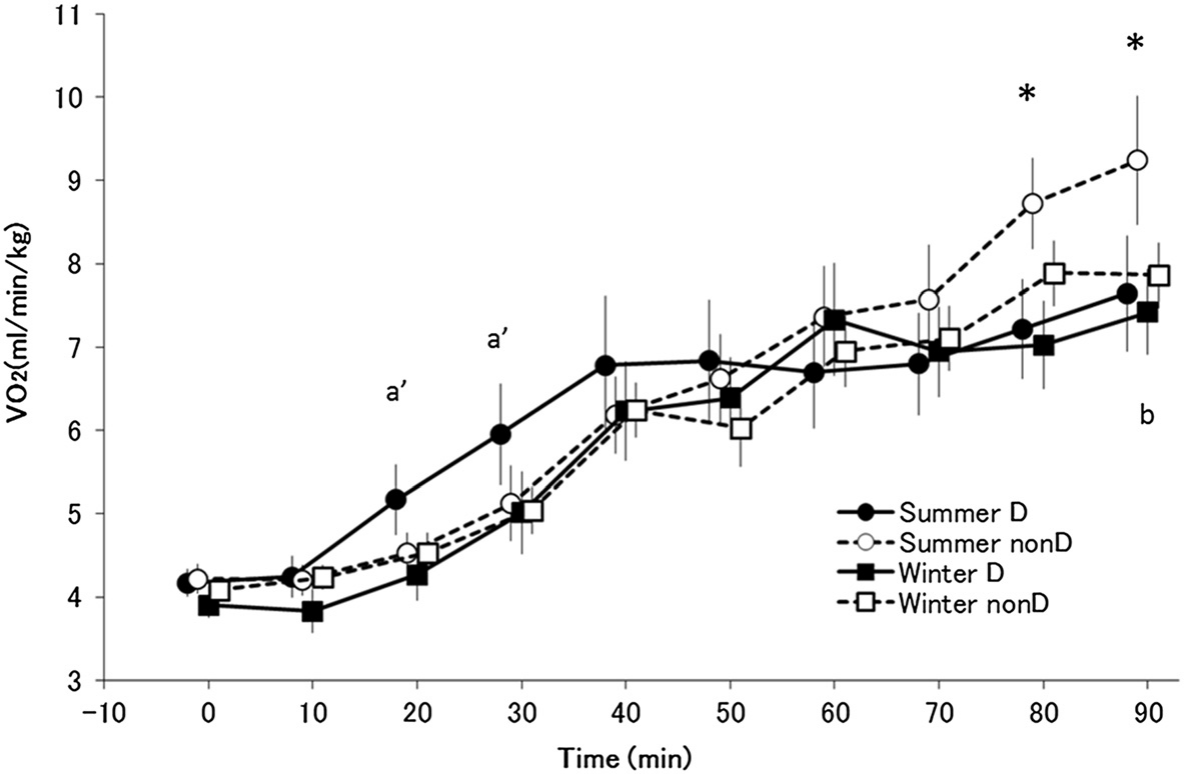
**Oxygen consumption.** Changes in oxygen consumption (mean ± standard error) in summer D (●; *n* = 7), summer non-D (○; *n* = 8), winter D (■; *n* = 7), and winter non-D (□; *n* = 8) groups. * *P <* 0.05 compared with summer D (●) and summer non-D (○), ^a^*P <* 0.05, ^a′^*P <* 0.1 compared with summer D (●) and winter D (■), and ^b^*P <* 0.05, ^b′^*P <* 0.1 compared with summer non-D (○) and winter non-D (□). In a *post hoc* test, haplogroup D oxygen consumption tended to be lower in winter than in summer 20 and 30 minutes after the start of exposure ( *P <* 0.1). In addition, in summer haplogroup D subjects had significantly lower oxygen consumption than non-D subjects at 80 and 90 minutes ( *P <* 0.05). Non-D oxygen consumption was significantly lower in winter than in summer 90 minutes after the start of exposure ( *P <* 0.05)

The main effect of time was significant (*F*_(9,117)_ = 44.815, *P* < 0.005) for oxygen consumption (Figure 3). There was also a significant interaction between group, season, and time (*F*_(9,117)_ = 2.57, *P <* 0.005).

In a *post hoc* test, oxygen consumption of haplogroup D subjects tended to be lower in winter than in summer 20 and 30 minutes after the start of exposure ( *P <* 0.1). In addition, in summer haplogroup D subjects had significantly lower oxygen consumption than non-D subjects at 80 and 90 minutes ( *P <* 0.05). In the non-D subjects, oxygen consumption was significantly lower in winter than in summer 90 minutes after the start of exposure ( *P <* 0.05).

### Mean skin temperature

**Figure 4:**
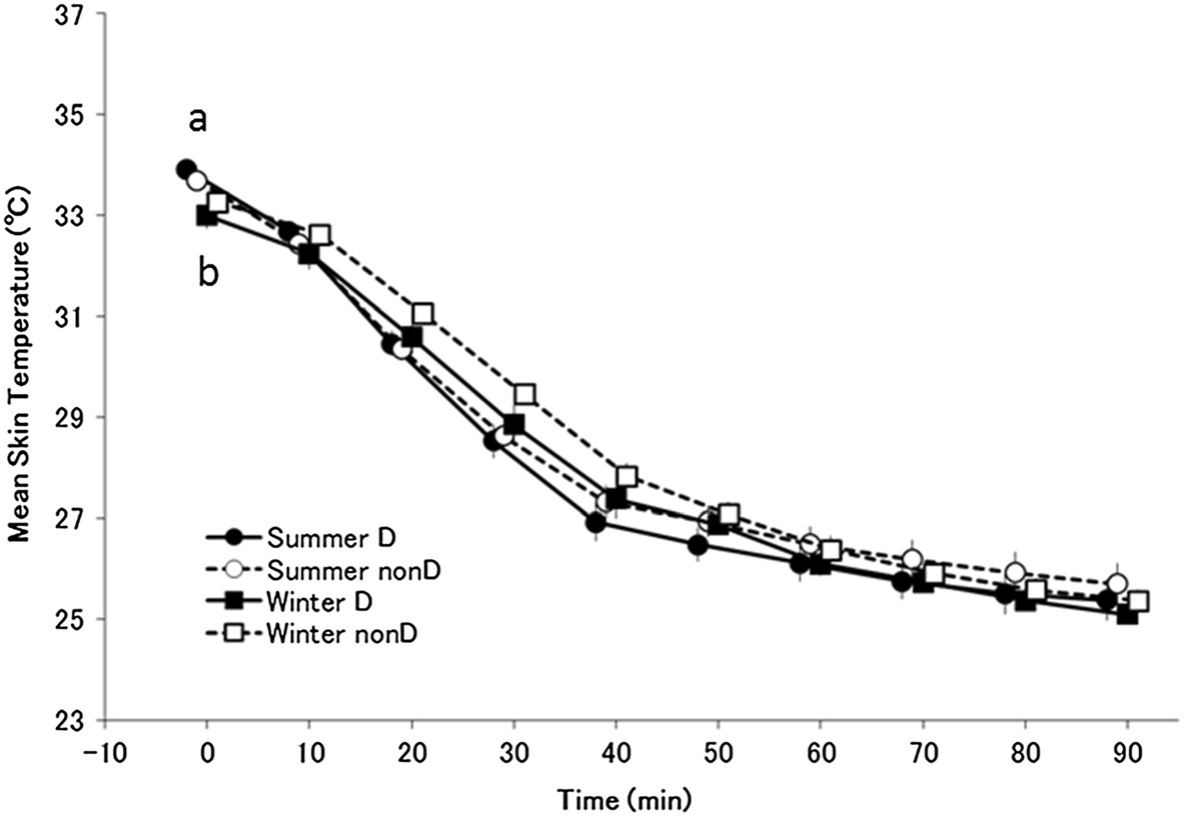
**Mean skin temperature.** Changes in mean skin temperature (mean ± standard error) in summer D (●; *n* = 7), summer non-D (○; *n* = 8), winter D (■; *n* = 7), winter non-D (□; *n* = 8) groups. ^a^*P <* 0.05 compared with summer D (●) and winter D (■), and ^b^*P <* 0.05 compared with summer non-D (○) and winter non-D (□). In a *post hoc* test, skin temperature at rest prior to cold exposure was lower in winter than in summer in both haplotype groups, but no other significant differences were observed

The main effect of time was significant (*F*_(9,117)_ = 1424.95 *P <* 0.001) for mean skin temperature (Figure 4). There was a significant interaction between season and time (*F*_(9,117)_ = 8.65, *P <* 0.001). There was no interaction among group, season, and time ( *F*_(9,117)_ = 1.81, p = 0.074). In a *post hoc* test, skin temperature at rest prior to cold exposure was lower in winter than in summer in both haplogroups.

### Rectal temperature and oxygen consumption during cold exposure

**Figure 5:**
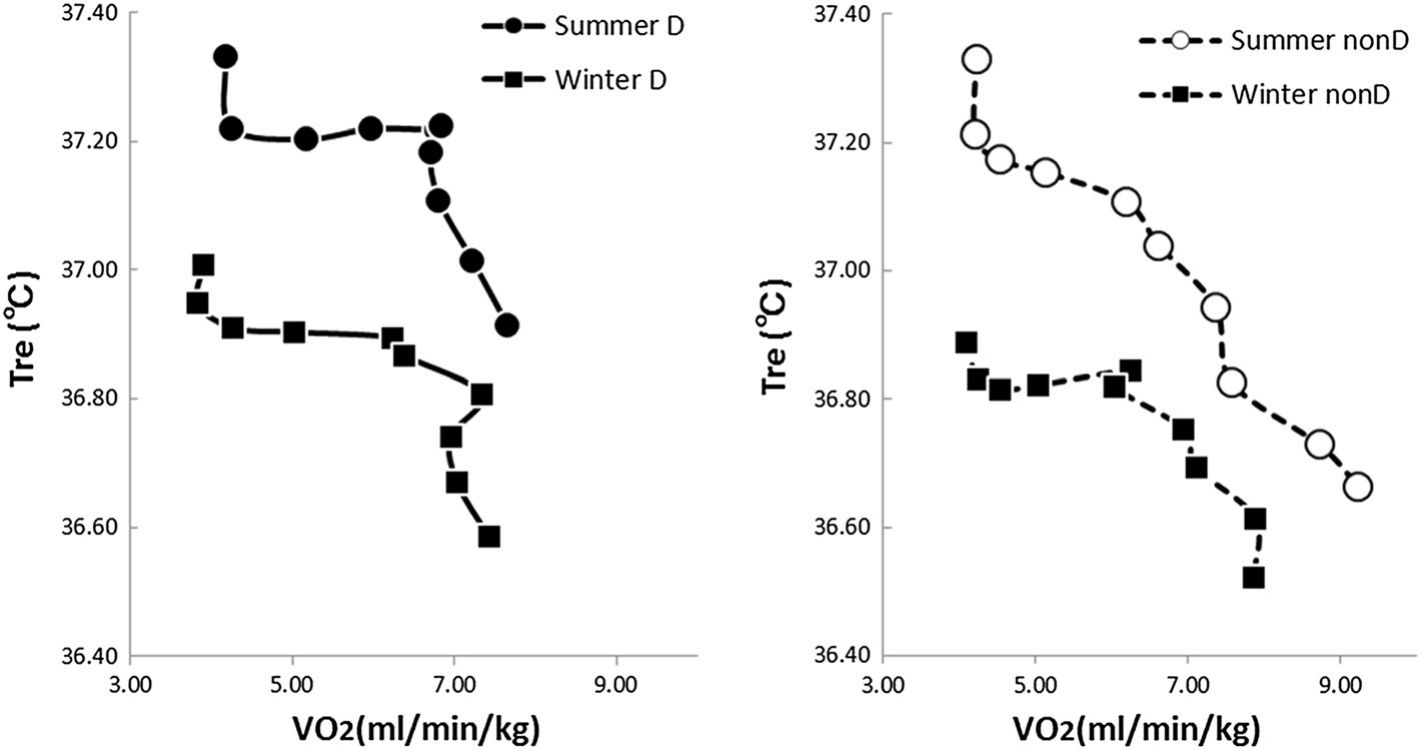
**Rectal temperature and oxygen consumption.** Rectal temperature and oxygen consumption during cold exposure in summer D (●; *n* = 7), summer non-D (○; *n* = 8), winter D (■; *n* = 7), and winter non-D (□; *n* = 8) groups. Similar results were seen in the summer and winter D groups and the winter non-D group. Only in the summer non-D group did metabolism increase as rectal temperature dropped, but rectal temperature continued to drop

The haplogroup D subjects showed a similar pattern of changes in summer and winter for rectal temperature and oxygen consumption (Figure 5). In the non-D subjects, rectal temperature decreased irrespective of increasing oxygen consumption in summer, but rectal temperature change showed a similar pattern to that shown by haplogroup D subjects in winter.

### Respiratory exchange ratio

**Figure 6:**
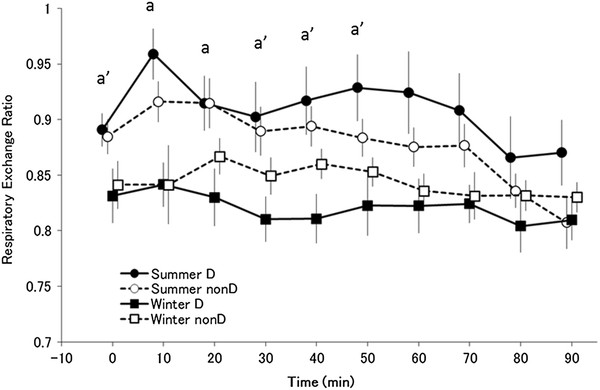
**Respiratory exchange ratio.** Changes in respiratory exchange ratio (mean ± standard error) in summer D (●; *n* = 7), summer non-D (○; *n* = 8), winter D (■; *n* = 7), and winter non-D (□; *n* = 8) groups. ^a′^*P <* 0.1, ^a^*P <* 0.05 compared with summer D (●) and winter D (■). In a *post hoc* test, respiratory exchange ratio in the haplogroup D was significantly lower in winter than in summer 10 and 20 minutes after the start of exposure and tended to be lower at 0, 30, 40, and 50 minutes ( *P <* 0.1)

The main effects of time (*F*_(9,117)_ = 9.666, *P <* 0.001) and season ( *F*_(1,13)_ = 6.694 *P <* 0.05) were significant for the respiratory exchange ratio (Figure 6). There was a significant interaction between season and time (*F*_(9,117)_ = 2.512, *P <* 0.05). More specifically, the respiratory exchange ratio was lower in winter than in summer. In a *post hoc* test, the respiratory exchange ratio in the haplogroup D subjects was significantly lower in winter than in summer 10 and 20 minutes after the start of exposure and tended to be lower at 0, 30, 40, and 50 minutes ( *P <* 0.1).

### Distal skin temperature

**Figure 7:**
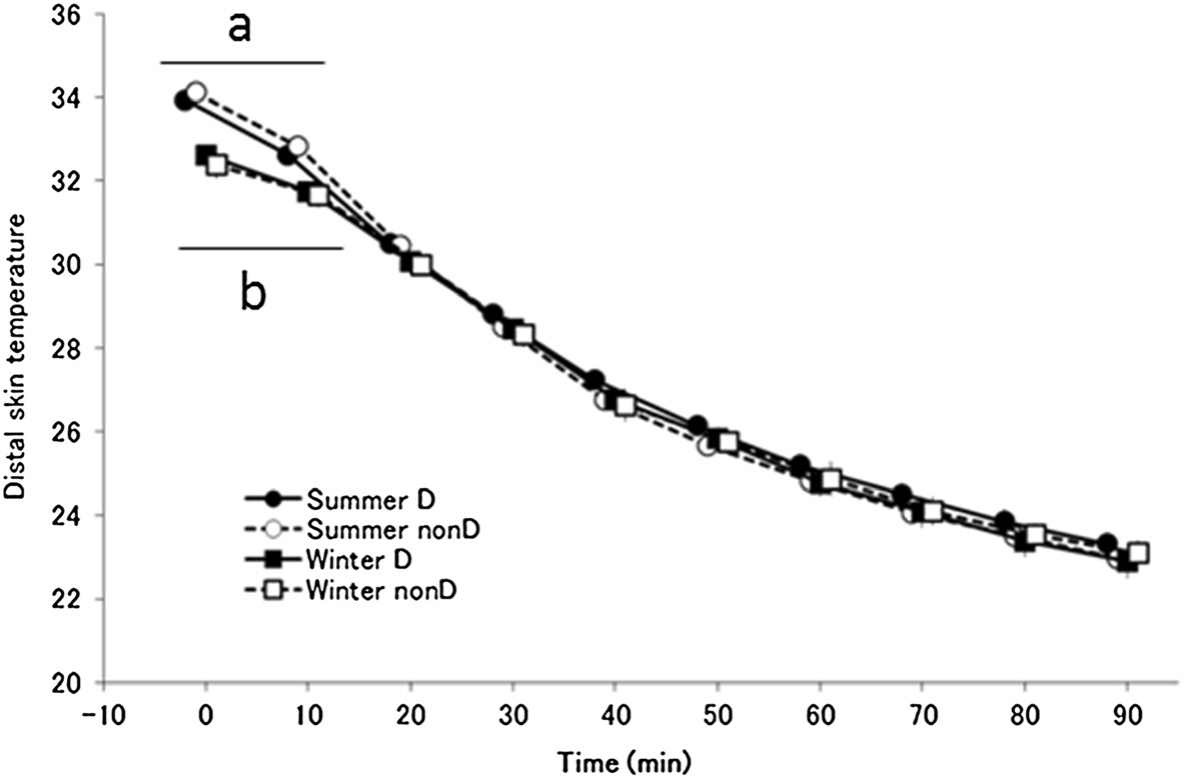
**Distal skin temperature.** Changes in distal skin temperature (mean temperature of the forearm, back of the hand, dorsal side of the foot, and thigh corrected for body surface area; mean ± standard error) in summer D (●; *n* = 7), summer non-D (○; *n* = 8), winter D (■; *n* = 7), and winter non-D (□; *n* = 8) groups. ^a^*P <* 0.05 compared with summer D (●) and winter D (■), and ^b^*P <* 0.05 compared with summer non-D (○) and winter non-D (□). In a *post hoc* test, the mean distal skin temperature in both haplotype groups was significantly lower in winter than in summer at 0 and 10 minutes ( *P <* 0.05), but no differences were seen after that

The main effect of time was significant (*F*_(9,117)_ = 3381.677, *P <* 0.001) for distal skin temperature (mean temperature of the forearm, back of the hand, dorsal side of the foot, and thigh corrected for body surface area; Figure 7). There was a significant interaction between season and time (*F*_(9,117)_ = 11.714, *P <* 0.001), and a tendency towards an interaction among haplogroup, season, and time ( *F*_(9,117)_ = 3.427, *P <* 0.1). Mean distal skin temperature in both haplotype groups was significantly lower in winter than in summer 0 and 10 minutes after the start of cold exposure, but no differences were seen after that.

No significant differences were seen in any other index (blood pressure, subjective evaluation).

## Discussion

The present study aimed to elucidate the relationship between seasonal acclimatization and haplogroup difference, with better cold tolerance defined as a smaller increase in energy metabolism in response to a decrease in rectal temperature. Statistical analyses suggested that the haplogroup, season, and duration of exposure influenced both changes in rectal temperature and increases in oxygen consumption due to cold stimulation. More specifically, the results suggested that genetic factors play a part in changes in rectal temperature and the increase in oxygen consumption, and these may be important associations.

The decrease in rectal temperature in summer was smaller in the haplogroup D group than in the non-D group, but no differences were seen between groups in winter (Figures 1 and 2). The thermoregulation response possibly changed due to seasonal cold acclimatization.

In a previous study, we reported that haplogroup D subjects showed strong tolerance to cold exposure in summer, with a small increase in energy, a small decrease in rectal temperature, and high NST in the body core [39]. In winter, haplogroup D subjects showed the same degree of decrease in rectal temperatures as in summer, but a tendency towards smaller energy consumption 20 and 30 minutes after the start of exposure compared with summer (Figure 3). In haplogroup non-D subjects, the decrease in rectal temperature in winter was the same as in haplotype D subjects and was smaller than the decrease in summer, and oxygen consumption was lower in winter than in summer 90 minutes after the start of exposure (Figure 3). Although the time period varied between groups, oxygen consumption was found to decrease in winter. These results provide an explanation for the type of seasonal cold acclimatization shown by haplogroup D subjects. The lack of difference in oxygen consumption 90 minutes after the start of exposure agrees with reports of no change in thermogenesis [16-18], and the decrease in oxygen consumption 20 to 30 minutes after the start of cold stimulation agrees with other studies that report a delay in shivering and a decrease in thermogenesis [13,14]. In contrast, oxygen consumption in non-D subjects was lower in winter than in summer 90 minutes after the start of exposure, which agrees with reports of a decrease in thermogenesis [2].

Cold acclimatization has been explained as enhanced insulation function and a change in metabolism. However, results for the distal skin temperature did not show a seasonal difference in the heat loss suppression response (Figure 7). Furthermore, rectal temperature decreased in winter, suggestive of acclimatization that is similar to isolative hypothermic adaptation in both group. However, a characteristic of haplogroup D subjects is a significantly lower respiratory exchange ratio in winter than in summer throughout the transition (Figure 6). Haplogroup D subjects may metabolize more lipids in winter than in summer, suggesting involvement of NST by lipid metabolism. For Japanese people, cold acclimatization has been reported to metabolize lipids better in winter and they also have a higher basal metabolic rate in winter [43]. In addition, thyroid hormone increases in winter for increased NST [44]. In association with these observations, although individual differences exist, brown adipose cells are generally more active in winter than in summer [31]. As seen by the decrease in the respiratory exchange ratio in haplotype D subjects during the first half of cold exposure in winter, it is possible that brown adipose cell activity begins increasing immediately after exposure, leading to an increase in NST from brown adipose cells that precedes ST. This hypothesis suggests that metabolism in mitochondria as a base may come to depend on lipids, irrespective of ST and NST. In either case, the amount of heat is larger in metabolism of lipids, indicating that efficient energy consumption occurred. The characteristic of haplogroup D individuals being good at metabolizing lipids may be indirectly related to a resistance to obesity and lifestyle-related diseases in this haplotype [45]. In other words, these results suggest that the type of seasonal cold acclimatization shown by haplogroup D is based on suppressing heat loss and is more dependent on lipids. Rather than ST, increased activity of brown adipose cells leads to more efficient NST. As a result, oxygen consumption decreases during the initial stage following the onset of cold stimulation (20 to 30 minutes), more energy is saved overall compared with summer, and rectal temperature is maintained. Haplogroup D people may have a type of seasonal cold acclimatization that relies on more efficient metabolism.

Acclimatization in haplogroup non-D may rely more on insulation compared with haplogroup D. If NST in brown adipose cells increased, the decrease in respiratory exchange ratio would also be large in the haplogroup non-D. However, the decrease in haplogroup non-D was smaller than that observed in haplogroup D subjects, and there was no significant seasonal difference (Figure 6). In contrast to haplogroup D, there is little variation in the respiratory exchange ratio, so variation in lipid metabolism would also be expected to be small. Haplotype non-D subjects reduce their core body temperature and suppress the loss of heat from the body surface, thereby suppressing the decrease in rectal temperature as well as the rise in thermogenesis. This is suggestive of hypothermic/isolative adaptation cold acclimatization. These results agree with previous studies reporting lower skin and core body temperatures [13,16] and a decrease in thermogenesis in winter [2,13-15]. This is the most frequently reported type of cold acclimatization.

The above results suggest that the hypothesis for cold tolerance in summer is correct from the perspective of maintaining rectal temperature. That is, genetic effects become apparent from differences in latent thermoregulation capabilities to respond to cold stimulation that seem novel during this time. The difference in cold tolerance in winter may be reduced, because the effects of seasonal cold acclimatization are comparatively larger in the non-D group than in the D group. This is the first study to demonstrate different forms of acclimatization in different haplotypes. More specifically, it is possible that haplogroup D people use a more metabolic cold acclimatization method, while haplogroup non-D people use cold acclimatization that relies on suppression of heat loss, resulting in a reduction in the difference in cold tolerance in winter. This suggests that variations in cold tolerance responses and types of seasonal cold acclimatization are related to mtDNA polymorphism and are influenced by genetics. In addition to differences in experimental conditions and seasonal factors, the lack of consistency with results from previous studies may be due to the existence of physiological polytypism among populations, with some relying on metabolism and some relying more on insulation.

In future studies, a genome-wide analysis approach is needed to quantify brown adipose cells involving basal metabolic rate or thyroid hormone and examine other genetic factors in addition to mtDNA polymorphism, as well as a larger sample size to enable statistical analysis. In particular, it is necessary to continue accumulating physiological and anthropological data in order to determine whether the group differences observed in the present study were the result of functional differences due to mtDNA polymorphism or due to another factor, such as population structure or the hitchhiking effect. These findings are probably important for discussions of physiological anthropology.

## Conclusions

Inter-group differences in rectal temperature and oxygen consumption are seen in summer but not in winter. This may be because cold tolerance is supplemented by seasonal acclimatization. The change in the respiratory exchange ratio suggests that the haplogroup D changes metabolism more, while haplogroup non-D rely more on insulation. This may mean that there is a relationship between mtDNA polymorphism and physiological polytypism, with a tendency towards either metabolic adaptation or isolative adaptation within population.

## Abbreviations

mtDNA, mitochondrial DNA; PCR, polymerase chain reaction; ST, shivering thermogenesis; NST, nonshivering thermogenesis.

## Competing interests

The authors declare that they have no competing interests.

## Authors’ contributions

TN and SW contributed to the design of the experiments. MM and YN contributed to data collection and analysis. YH and RK contributed to the genetic analysis. All authors read and approved the final manuscript.
